# The Treatment of Phthisical Cough

**Published:** 1880-03

**Authors:** 


### THE TREATMENT OF PHTHISICAL COUGH.

At the last meeting of the Ohio State Medical Society, Dr. Robert Bartholow read a paper on the Treatment of Phthisis, from which we extract the following remarks as to the means of relieving cough, often one of the most troublesome questions with which we have to deal in the treatment of phthisical cases.

"As cough prevents sleep, destroys rest at all times, and is exhausting, patients are clamorous in their demands for relief. The prescription of hydrobromic acid and spirits chloroform, proposed by Fothergill, is rather disappointing; gargling the throat with solution of potassium bromide allays the reflex excitability of the fauces, and thus lessens cough somewhat. But there are no efficient substitutes for the preparations of opium, and they must usually be prescribed when the cough is severe and persistent. The most generally useful of these is codeia, which is about one-half the strength of morphia. The superior virtues of codeia as a remedy for cough are these: it has a selective action on the pneumogastric nerve, allaying irritability of its end-organs; it is calmative and hypnotic, and as compared to morphia, is less excitant and less nauseant. Codeia may enter into extemporaneous combinations with atropia and other agents just as morphia does.

"Apomorphia now and then succeeds admirably in allaying cough, but it is useful in the *rôle* of nauseating expectorants, and is therefore not suitable in phthisical cases. When an irritable stomach co-exist with a teasing cough, oxalate of cerium acts surprisingly well. On the other hand, for the arrest of the reflex vomiting of phthisis which accompanies the cough, there is no remedy comparable to strychnia; when there is profuse expectoration, rather fœtid in character (bronchiectasis), and indeed in ordinary cases with free expectoration, carbolic acid is often very beneficial. It may be given in cherry laurel water in the dose of half a grain three times a day. Besides its influence over cough in these cases, carbolic acid reduces the abnormal temperature, checks the sweating and improves the bodily condition in general. In the more chronic cases of phthisis, with a less range of daily temperature and scanty expectoration, iodide of ammonium is specially effective. It is useful to combine arsenic with it, or it may be given in a mixture of wine of tar."

